# Animal Welfare Issues in Capture-Based Aquaculture

**DOI:** 10.3390/ani11040956

**Published:** 2021-03-30

**Authors:** Uthpala Chandararathna, Martin Hugo Iversen, Kjetil Korsnes, Mette Sørensen, Ioannis N. Vatsos

**Affiliations:** 1 Faculty of Biosciences and Aquaculture, Nord University, Universitetsalléen 11, 8026 Bodø, Norway; uthpala.chandrarathna@nord.no (U.C.); martin.h.iversen@nord.no (M.H.I.); kjetil.korsnes@nord.no (K.K.); mette.sorensen@nord.no (M.S.); 2 BioVivo Tech AS, Stobjørnen 17, 8029 Bodø, Norway

**Keywords:** fish, capture-based aquaculture, welfare, welfare indicators

## Abstract

**Simple Summary:**

Aquaculture is about farming aquatic animals in certain facilities, in order to meet the global demand for aquatic animal products. However, reproduction and raising of all commercially important aquatic animals under farming conditions are not always feasible or cost-effective. Thus, for some fish and shellfish species, a system that relies on the collection of wild individuals, at various life stages and rearing in controlled facilities, has been employed. These animals, however, are not products of a lengthy domestication process and thus are not used to the artificial environment of the fish farms. The way they respond to the various stressful conditions might be different, compared to the domesticated fish. Therefore, we have to make sure that we use the appropriate rearing methods, for the entire time, so that their impact on the fish welfare is minimized. Apart from the moral obligations, this will also increase the profitability of the activity.

**Abstract:**

Capture-based aquaculture (CBA) represents a type of intensive aquaculture production system for some economically valuable fish species, such as bluefin tuna (*Thunnus thynnus),* eel (*Anguilla* spp.) and Atlantic cod (*Gadus morhua*). In CBA, fish are captured from the wild in certain periods of the year, and following a recovery phase, they are kept in rearing facilities for a period of time, until they reach the market size. In this case, the fish are wild and have not gone through domestication like other fish species that are reproduced and farmed under the established farming systems. Therefore, these fish are not genetically adapted to live under the intensive farming conditions, and thus their welfare may be compromised in different manners compared to their domesticated counterparts. This review presents an overview of the current situation of CBA, while focusing on the assessment of fish welfare in CBA. The most commonly used fish welfare indicators will be discussed in relation to the different stages of CBA.

## 1. Introduction

Aquaculture is considered as the fastest growing sector in food industry, representing 46% of global fish production in 2018. As the supply of capture fisheries remains almost static from the 1980s, aquaculture supplies significant quantities of fish worldwide [1]. According to historical evidence, the beginning of aquaculture dates back to 4000 years ago, when common carp was cultured in China, as a result of population settlements [2,3]. Initial aquaculture practices, solely depended on wild seed, for example, from gravid females or early life stages. But unpredictability of wild seed collection often prevented large scale aquaculture practices until the development of hatchery techniques, which produce reliable number of seeds.

Aquaculture can be operated as a ‘closed cycle’ system, where the cultured aquatic animals complete their entire life cycle, or as hybrid systems, as culture-based fisheries and capture-based aquaculture [4]. Nevertheless, aquaculture methods are highly diversified according to the species cultured and the production systems used. Capture-based aquaculture (CBA) can be identified as a recently intensified aquaculture production system. Even though the term “capture-based aquaculture” is relatively new, as it is was introduced in 2004, CBA has been practiced locally for many years in many countries through traditional means, for a variety of freshwater and marine fish and other aquatic invertebrate species [5]. The strategy used in CBA can be described as a hybrid approach between capture fisheries and aquaculture. In simple definition, CBA is harvesting aquatic animals of early life stages to adults from the wild environment (often referred to as ‘seed’ material) and culture or fatten them in grow-out facilities prior to marketing. Tuna ranches and eel farms are two of the increasingly growing and highly profitable, CBA practices worldwide. The history of Atlantic cod (*Gadus morhua*) CBA in Norway goes as far back to the early 1880s, when Norwegian sailing vessels landed in England carrying live cod. Catfish aquaculture in Vietnam, shrimp culture in Bangladesh, giant snakehead traditional cage culture in Cambodia and milkfish farming in Philippines are good examples of where CBA is practiced regionally in Asia [5,6].

Welfare of the farmed fish has already become a critical issue for the farmers and the society in general, but as CBA uses wild fish and not domesticated, there are particular concerns about any specific issues regarding the welfare of these fish, that are kept under these conditions for a period of time. Therefore, the present review, will focus on the stages of capture-based fish farming and the particular welfare issues related to them. It should be noted though, that any ethical issues regarding the exploitation of wild animals are beyond the scope of this review.

## 2. Overview of the CBA Practices

### 2.1. Main Phases of CBA

#### 2.1.1. Capture, Transport, Short Term Storage and Recovery Phase

In this phase, certain life stages, depending on the target species, are captured as live seed. The capture methods include many active and passive fishing gears, and care is taken to collect the animals with the least harm (Table 1). Then, the animals are transported to short-term storage facilities, where they are sorted according to the need. For example, many live seed gathered from the wild, need to go through a recovery phase, to recover from the catch stress and to be weaned before the aquaculture phase begins (Table 1). Strategies used for recovery and weaning are species specific, but most share the common methods, like short-term starvation, feeding of wet foods, especially designed tank/pens etc. [5,7].

#### 2.1.2. Aquaculture Phase

In the aquaculture phase, the recovered and healthy animals are cultured throughout a predetermined period, using suitable husbandry practices such as housing and feeding. When animals reach the market size, they are slaughtered and marketed as fresh or processed products. However, these components of CBA methodology are largely dependent on the targeted species (Table 1).

### 2.2. Advantages and Disadvantages of CBA

#### 2.2.1. Advantages

##### Ability to Culture Species Which Cannot Be Bred in Captivity

With the recent advancement of the aquatic sciences, we can breed many commercially valuable aquatic species under captivity. This helps to ensure the supply of reliable number of larvae or juveniles on a large scale in aquaculture facilities and to maintain regular production cycles. However, there are species-specific challenges, such as environmental requirements and complex migratory patterns for captive breeding. For example, a field study on oceanic spawning of the Japanese eel (*Anguilla japonica*) and the giant mottled eel (*Anguilla marmorata*) revealed that after migrating from freshwater habitats to the ocean, many oceanographic, geological and lunar cues are used by the eels for spawning [13]. Bluefin tuna (*Thunnus thynnus*), is known to perform extensive seasonal reproductive migrations, which are biologically important for sexual maturation between feeding and spawning grounds [14]. Such reproductive behaviors are almost impossible to stimulate under captive conditions, hence the eel and tuna farming have to depend mainly on the seed collected from the wild, although promising advances in the farming of both species might change this in the future.

##### The Low Cost Approach

Hatchery production of larvae/juveniles for commercially valuable fish species often requires high cost and special knowledge. The hatchlings should be reared up to certain sizes before stocking, which requires nursery facilities and feeding special larval feed. Furthermore, growing hatchlings to marketable size can be a long-term process depending on the species. In contrast, when the larvae/juveniles are collected from the wild, they are grown to a certain size and have often passed the critical life stages. Using them for aquaculture purposes can be cost effective due to the reduced cost for nursery care. Another benefit of growing out wild seeds, is the shortening of grow-out or fattening period. Evaluation of value adding properties of Atlantic cod CBA, stated some key parameters such as increased yield, improved quality characteristics, compared to wild-caught cod and size related prices, that can increase the revenues in CBA. In cod farming, the production cycle to get an average weight of 4 kg fish is about 24–36 months and due to early maturation and size limitations, it may result in smaller size cod [11]. Moreover, the cod larvae need to be fed with live feed, followed by special larval diet, and longer production cycles are often prone to disease outbreaks, which lead to heavy mortalities. Longer production cycle indicates high cost and added uncertainty of the final yield, due to losses at different life stages. On the other hand, in cod CBA, and depending on the stocking size, about 4 to 5 kg cod can typically be produced in 6 to 8 months with feeding. In CBA, the cost is mostly related to the catching cost, which can be high, particularly if a long time is required to capture the fish. The feed cost includes both the wet feed and the dry feed. However, previous analyses of cod CBA suggest that cod CBA is still economically preferable, compared to traditional cod farming, despite the fact that the prices and the quality of the farmed cod are often better [11,15].

##### Importance in Rural Communities

Fish being one of the most important protein sources, contribute to 17% of animal protein consumption in many rural communities in developing countries [16]. In Africa alone, fish has become the main protein and micronutrient source for about 200 million of its population. However, artisanal marine or freshwater fisheries are not reliable to provide adequate supply of fish/shellfish. Development of CBA is ideal in rural areas, since CBA facilities could be installed with lower number of infrastructures and require less specific technical knowledge than hatchery-based aquaculture. For example, wild-caught catfish *Clarias* aquaculture in the western Cameroon highlands supports the rural communities as a livelihood to improve their living standards [7]. Furthermore, in Philippines, while most large-scale aquaculture farms rely on hatchery bred milkfish fry to sustain milkfish aquaculture, many small-scale farmers in rural areas still highly depend on milkfish fry collected from the wild [12,17].

##### Use of Low-Value/Trash Fish in CBA

The use of low-value/trash fish in CBA may have some advantages in some cases [18]. Most of these fish are used either for the production of fishmeal, or for human consumption. However, a large proportion is also used for CBA. For example, it was estimated that in 2002, up to 180,000 t were used for the production of about the same mass of Pangasiid catfishes, in Viet Nam [7]. The advantages of using these fish are the low cost, the feeding efficiency and the possibility that small-scale farmers can produce the feeds on site [18]. On the other hand, apart from the irregular supply, the main concern is the increased waste production, due to the discarding of the undigested parts into the environment.

#### 2.2.2. Disadvantages

##### Overexploitation of Wild Fish Stocks

Over exploitation of wild seed for CBA can be devastating to wild fish stocks by lowering the natural recruitment of sexually mature adults. For example, the catch amount of European glass eel displayed a decreasing rate over the last few decades due to severe overexploitation, forcing governments to limit the collection of glass eels, as the European Council Regulation EC No 1100/2007 describes. Even though there is no direct scientific evidence about overexploitation, wild seed collection for Pangasius catfish CBA in Vietnam and Cambodia is controlled under government regulations [7].

##### Non-Targeted Animals–Bycatch

For CBA, in many species usually the target life stage is larvae or juveniles/sub adults, which are small in size. Therefore, the fishing nets used for seed collection have small mesh sizes, which leads to increased bycatch [7]. Furthermore, on some occasions, such as larval drift or migration, the juveniles and larvae of different species tend to do shoaling or come together, and this can increase the chances of bycatch of untargeted species. Fishing gears, for example ‘fyke’ nets which are set in lagoons for eels can cause bycatch by trapping unwanted organisms (especially freshwater turtles) [5,7]. In catfish fry collection with ‘dai’ nets, bycatch of other cyprinids could occur as high as 75–90% [7]. Therefore, CBA induced excessive bycatch can be detrimental for natural populations and endangered species. An additional problem, not only related to CBA, is that very often, some of these nets are lost or abandoned in the sea, becoming what we call ‘ghost nets’. These nets can have serious environmental consequences to the wild aquatic populations, as various animals can still be trapped in them for long periods of time.

Regardless of the capture method, when wild fish are captured, they encounter various stressors, from the contact with the fishing gear, to handling and the final confinement. The consequences are not always easy to assess, as the duration of the explosion and the cumulative effect and the physiology of the specific fish species can affect the outcome. This is issue is further discussed in Section 3.2.1.

##### Ecosystem Degradation and Pollution

Ecosystem degradation is a common issue with many techniques used in marine and freshwater capture fisheries. Some fishing gears like bottom trawls can destroy the ocean bottom and dislocate the sessile organisms. Also, fishing methods used to capture fish in coral reef areas such as groupers can cause breakage of reef, block the crevices etc., which can be harmful to the reef, and increase the habitat loss of reef dwelling organisms [5,7].

##### Transmission Risk of Diseases

Intensive aquaculture practices with high stocking densities often make fish susceptible to diseases, which can cause huge economic losses. Previous incidents have proved that, disease transmission from the wild fish to the farmed fish and vice versa is possible [19]. In ‘closed cycle’ intensive aquaculture systems such as for salmonids in Norway, the hatchery reared fish stocks are often checked for different pathogens, and vaccination against some diseases is carried out. But, in CBA, the stocked fish are directly taken from the wild, and pathogens may be introduced to farming environment through carriers from the wild, and thus there is a risk of transmitting diseases to other vulnerable host species that are farmed in close proximity. Moreover, unlike in many commercially farmed species in closed cycle aquaculture, for some species cultured in CBA, as for instance yellowtail tuna and during certain stages of cod rearing, low-value fish/trash fish are extensively used as feeds, which consist of either bycatch or smaller fish. Usage of wild caught low-value/trash fish as feeds, could also be another way to transfer pathogens from the wild to farming areas [6]. Thus, assessment of wild fish stocks for the presence of any disease is important to reduce the transmission risk of pathogens to cultured species [19].

## 3. Welfare Requirements and Effective Welfare Management in CBA

### 3.1. Fish Welfare in Aquaculture

According to Stien et al. [20], animal welfare can be defined as the quality of the life as perceived by the animal itself. Animal welfare has been assessed in many aspects such as function-based, nature-based, and emotion-based [20]. In a well-managed farming system, with good welfare, the animal should be able to perform its physiological functions, such as growth and its natural behaviors adequately. In addition, the environment should imitate the natural habitat as much as possible. Animal’s welfare needs can be categorized as ultimate or proximate needs. Ultimate needs are the essential for its survival needs, such as respiration and nutrition, while proximal needs are defined as the needs, which are important to improve the animals’ ability to succeed in the long term, for example behaviors like jumping in salmon, or playing in young animals improve body strength and control [21,22]. The fulfillment of the needs induce satisfaction, and unsatisfied needs can cause frustration or suffering in animals [21,22,23,24,25]. Thus, creating opportunities for ‘positive emotional states’ is considered very important and should be taken into account when assessing the welfare of the animals.

Compared to farmed terrestrial animals, the issue of welfare in fish farming has received increased attention only in past two decades, as an increasing amount of publications and reports have demonstrated that fish are conscious about the surroundings, can suffer and feel pain [23]. Experimental behavioral studies of interactive learning of fish have proven that fish are capable of understanding their environment to make mind maps, based on learning and executing learned behaviors. For instance, according to McGregor et al. [26] fighting behavior analysis of Siamese Fighting fish (*Betta splendens*) showed that they gather information of peers’ fighting abilities through observational learning and use to modify subsequent actions such as choosing to fight with weaker individuals which give higher chance to win. In another study, Kohda et al. [27] explored the ability of fish to be self-aware by using the mark test, observed behavioral responses in cleaner wrasse (*Labroides dimidiatus*) that fulfilled the criteria. The authors could not conclude with certainty whether the fish were self-aware or not, but the findings raised serious questions. Presence of nociceptors indicates the fish’s ability to detect and respond to noxious stimuli [28]. Glucocorticoid receptors and serotonergic activation due to social stressors, suggest that fish can experience the effects of stress [29]. Thus, we can suggest that fish possess cognitive abilities and suffering and the capacity for suffering, or distress caused by stressors can disrupt their wellbeing. Furthermore, in a profit-based production system, negative feelings induced by inadequate welfare may affect animal’s growth and other physiological functions and cause economical losses [21,30].

### 3.2. Welfare Assessment in CBA

To assess whether the animal welfare needs are fulfilled or not, there should be a set of measurable standards. The concept of welfare indicators (WIs) was developed to evaluate the extent to which welfare relevant needs are met. Animal-based WIs are based on direct observations/measurements of the animal itself and environment-based WI can be classified as indirect and rely on the resources and environment related to the animal [20,21,31]. There are strengths and weaknesses of both type of WIs in welfare assessment. For example, animal based WIs indicate the actual conditions of animals, while environment based WIs can be used in risk assessment [21]. However, all WIs that have been studied extensively, cannot be used on site in the field, especially in fish farms, if for example, they require a laboratory, or specific equipment [21,32]. Therefore, it is important to select reliable WIs that are easy to use in fish farms. They should also be comparable and repeatable. In Noble et al., [32], these WIs are recognized as Operational Welfare Indicators (OWIs). OWIs in aquaculture are mainly categorized as animal based (individual and group based) and environment based OWIs [21,32].

In general terms, welfare of farmed animals often depends on the degree of domestication of the livestock animals [2]. Domestication of terrestrial livestock mammals for food production goes far back to approximately 9000 years ago, in 7th millennium BC according to radiocarbon evidence in archeological remains, as hunter-gatherers began to raise herds of sheep and goats [2,33]. Previous studies of domesticated animals reveal that there is an apparent dichotomy between domesticated animals and their wild counterparts in many ways [2]. For example, through selective breeding over many successive generations, domesticated animals have acquired certain genetic traits, such as faster growth rates and higher milk production as well as different behaviors, such as less fear to human handling and lower motivation for foraging [2,33].

Although early historical evidence of aquaculture practices in China, dated back 4000 years ago, the ‘blue revolution’ (i.e., the transformation of the traditional fish farming activity into the industrial mode of fish production that we have today) occurred in the mid-1960s. Today, about 622 aquatic species (including hybrids) are farmed worldwide [1], however there is limited information available about their domestication state, except for a few species. Teletchea and Fontaine [2], discussed a detailed classification of the fish domestication levels, which range from 1 to 5. According to this, fish at level 5 are considered as true domesticated fish, which are selectively bred to achieve specific goals, such as higher growth rate, or fillet yield. Example species include farmed Atlantic salmon (*Salmo salar*) and rainbow trout (*Oncorhynchus mykiss*). However, most fish species used in CBA, which complete a part of their life cycle in captivity, fall into the pre domestication phase indicated as levels 2 and 3.

There is scientific evidence, which suggests that stress levels may differ between domesticated and wild animals. In one study, offspring of wild and sea-ranched (domesticated) sea trout (*Salmo trutta*) were subjected to two standardized stressors: transfer into novel environment and predator exposure [34]. The blood plasma glucose levels, brain 5-HIAA/5-HT and DOPAC/DA ratios were found to be significantly higher in wild trout than in the domesticated trout following post stress experience. In another study, hematocrit and plasma cortisol levels of wild chinook salmon (*Oncorhynchus tshawytscha*) were significantly higher than hatchery reared chinook salmon in both low and high stocking densities [35]. In Jentoft et al. [36], effects of standard handling on growth and stress parameters were studied in non-domesticated Eurasian perch (*Perca fluviatilis*), a promising aquaculture candidate for commercial perch aquaculture, and compared to those of domesticated rainbow trout (*Oncorhynchus mykiss*). Although the authors admitted that comparison between the two different species is difficult, they noted that Eurasian perch exhibited higher post stress glucose and cortisol levels, after stressed, either once or repeatedly, when compared to the domesticated rainbow trout. Furthermore, evaluation of stress responsiveness on growth performance between the two species showed that stressed Eurasian perch had 35.4% lower mean body mass compared its non-stressed control, while the mean body mass difference was lower (22.8%) in rainbow trout suggesting the increased stress levels may lead to high energy consumption in non-domesticated Eurasian perch [36]. Hence, it is suggested that the differences in stress levels and following consequences in wild caught fish used in CBA need to be considered when assessing the overall welfare.

Considering the welfare assessment in CBA, we can identify three main phases: (a) capture phase, (b) recovery phase, and (c) aquaculture phase. These need to be assessed separately using specifically adapted OWIs (Table 2).

#### 3.2.1. Welfare Assessment in CBA during Capture Phase

In CBA, we can identify two main groups of animals whose welfare assessment is important to be discussed in this phase.

##### Welfare of the Target Species

There are several differences between commercial capture fisheries and capturing fish for CBA. Firstly, in commercial capture fisheries, the fish collected are either dead at collection time point, or alive, but will be slaughtered and processed soon after they are collected [37]. On the other hand, in CBA, the captured target fish should be alive and healthy. In addition, commercial fisheries, usually target fully grown adult fish and the bigger are usually the better. In CBA, the target fish at young life stages are also considerably smaller in size at capture, hence easily subjected to physical injuries. Thus, the fishing methods should not inflict lethal physical trauma to the fish during the capturing [7]. Furthermore, fish must be capable of recovering from any harm/injury that occurs during capturing. Here, both the fishing gears and the fishing time between the first encounter of the fishing gear and the collection, play an important role.

Modifications in the fishing gear, to induce less harm, and reduce the fishing time, can be used as preventive measures, and will improve the welfare in CBA during capturing. Cook et al. [38] provide an interesting review on the capture-related stress and possible mitigation measures. Although the review refers to commercial fisheries, some approaches can also be used in CBA. For example, in cod CBA, the fishing gears mostly used are Danish and Scottish seine nets. As a modification, a canvas lining has been added to the cod end to avoid physical injuries caused by pressure. Unlike in traditional fisheries, the hauling speed is much reduced during live cod capturing process to allow slow release of air from the gas bladder [7].

##### Welfare of the Non-Targeted Species

One main drawback mentioned in relation to CBA wild seed collection, is the sometimes excessive bycatch. These unwanted species will be discarded at fishing site, or at the landing site. However, unlike in commercial fisheries, in CBA, there is a high possibility that bycatch consists of young life stages of non-targeted species, as the fishing gears used have usually smaller mesh sizes. Fishing stress and physical damages can considerably lower the survival of these young non-targeted species, like larval stages and juvenile fish and other aquatic animals, even though they may be released to the wild afterwards. Milk fish wild seed collection is an example that produce high bycatch of juveniles of non-target species such as black tiger shrimp (*Peneaus monodon*), snapper (*Lutjanus fulviflamma*), anchovy (*Encrasicholina heteroloba*), tiger perch (*Terapon jarbua*) etc., which are potential marketable species in capture fisheries [17]. However, for most fishing methods that are currently being used, the status of bycatch is less studied. Therefore, the fishing methods should be adapted to lessen the bycatch to improve the welfare of non-targeted species [6,7]. For example, a simple modification of the entrance funnel of the fishing trap used to capture glass eels suggested by Lopez and Gisbert, appears to significantly reduce the capture of the non-targeted fish, without affecting the glass eel captures [39]. The capture of unwanted aquatic species is big issue in commercial fisheries in general, and therefore, various legislations in different parts of the world, as for example within the European Union, in Chile and in Norway, regulate the discard of any unwanted catches and the maximum allowed catch, aimimg to improve the fishing behaviour and to minimize the losses of non-targeted species.

#### 3.2.2. Welfare Assessment in CBA during Recovery Phase

In CBA, wild caught fish usually undergo a recovery phase before further handling or transport and transfer to aquaculture settlements. Recovery period is useful in restoring the physiology following the capturing stress and to acclimatize to life in a confined environment. Length of recovery periods could vary from a few hours to a few days according to species. Even though there is a lack of information about the recovery steps implemented for wild seed for many fish species used in CBA, considerable number of studies exist on the recovery from capturing for a few species. For example, after hauling and landing, the live Atlantic cod juveniles are placed in specifically designed net cages with a flat or taut bottom, at least 2.5 km from any farming site, for up to 24 h, to assist refilling the gas bladder and to recover the ruptures of the gas bladder resulted from the hauling [7]. In other cases, captured fish recover during the transportation to the rearing sites, as in the case of bluefin tuna [7].

At this stage of CBA, welfare assessment should be focused on the stress management related to fish handling and transport, which occurs in between the capture from the wild and the transfer to the aquaculture facilities. As discussed earlier, being non domesticated wild fish, handling, transport, and introduction to a confined environment can induce higher levels of stress in fish, which may reduce the immunity, making them vulnerable to diseases and ultimately leading to higher mortality rates. Therefore, it is suggested that the OWIs used in the recovery stage should be more connected to the health of the individual fish, for example the level and the type of injuries in fish (e.g., scale-loss, superficial injuries) and any sudden behavioral changes, such as disoriented swimming. These can give farmers a better insight of the condition of the fish prior to the start of any further operations. Furthermore, avoidance of lengthy transportation without a recovery period may enhance the survival of the fish at the beginning of aquaculture stage.

Laboratory Based Welfare Indicators (LABWIs) can be used at this stage, but also during the aquaculture phase. These indicators are not considered OWIs, as they require a laboratory. Some of them though, like plasma glucose levels, require simple equipment, and thus, they can be assessed on site. However, they are not recomemended for all circumstances and they are more appropriate for research purposes.

#### 3.2.3. Welfare Assessment in CBA during Aquaculture Phase

Fish welfare in the farming of fish extensively farmed worldwide, as for example salmonids, has received a lot of public attention, and studied quite extensively compared to CBA [21]. However, in CBA, a wide variety of fish are used, and since it is categorized as a marginal industry, research studies, or data related to the biology/physiology of most fish used in CBA are hardly available. Furthermore, some CBA practices are of interest to certain small geographical regions. Therefore, this knowledge gap prevents scientists from identifying welfare assessment needs, which can readily be used in CBA industry.

In CBA aquaculture, the length of aquaculture periods could be highly variable, according to the species but also according to the size at stocking for the same species. As an example, in tuna CBA, some farming sites in Japan, stock small specimens (150–500 g body weight) and rear for 3 to 4 years in net cages until fish reach the marketable size of 30 to 70 kg. But, in the Mediterranean area, tuna farming, stocked fish are larger than 6.4 kg, the legal minimum size set by ICCAT (International Commission for the Conservation of Atlantic Tunas), and thus larger individuals may be harvested in 2 to 6 months, while small ones are reared for up to 3 years [5]. Furthermore, many CBA practices, such for tuna and yellowtail, still depend on wet food, such as low -value/trash fish, in one or many occasions during the aquaculture phase [7], because wild fish, especially when the initial life stage used for stocking is older, without domestication practices still seek for their natural food sources rather than dry pellet or flake feed. Palińska-Żarska et al. [40] noted that domesticated Eurasian perch, larvae are characterized by lower activity of the digestive enzymes and lower expression of the genes encoding the digestive enzymes, a phenomenon probably related to the lower diversity of food offered in culture conditions. Furthermore, the amount of feed with respect to biomass is equally important. If the fish receive less feed, or feed of inappropriate quality, malnutrition will be observed, often accompanied by increased aggression and probably cannibalism, as it has been observed in glass eels and groupers [5]. Therefore, individual-based welfare indicators, especially growth-related OWI, such as condition factor, are important to analyze the quality and effectiveness of feed the farmer is using during live storage period. Adjustment according to species should be made, as for instance, the hepatosomatic index has been suggested as a representative indicator in cod [41].

Mortality is a common WI, and expressed as a cumulative number of dead fish in a relevant time period. Unlike in capture phase and recovery face in which duration is shorter, long term mortality could be as a group-based OWI during live storage/aquaculture phase in CBA. Real time mortality metric could be acquired by regular removal of dead fish from the rearing system, which can also easily be adapted as a farmer-friendly robust OWI in farming facilities [21,42]. It should be noted that as abnormally high mortality are also an indicator for disease outbreaks, removing dead fish regularly can help to decrease the possible sources of the disease.

Water quality degradation could be rapid and disastrous when uneaten low-value/trash fish accumulate. Therefore, frequent water quality analysis using selected environment based OWIs (e.g., dissolved oxygen, pH, nitrogen wastes, odor and color of the water) may prevent mortalities occurring due to poor water condition [21]. Considering the housing systems and the stocking densities in CBA, specific behaviors of fish, such as cannibalism, territorial behavior etc. are important. Stocking at appropriate densities and grading into similar stocking sizes may reduce the loss of fish due to cannibalism.

## 4. Conclusions

Farming of wild animals is a very controversial issue. Despite many ethical considerations, it is a commercial activity that can provide substantial income in certain regions. Thus, it is important to confirm that the welfare of these wild animals that are reared in captivity is not compromised. CBA is a hybrid aquaculture practice, consisting of collection of wild seed, and restocking and culturing them up to market size in man-made grow-out facilities. CBA is being practiced worldwide for many different fish species. Regarding the welfare assessment in CBA, further research is needed, as the significant differences in terms of physiology and behaviour between the domesticated and the wild fish might require different approaches. As in the case of domesticated fish, there is no single indicator that can provide enough information to sufficiently assess the welafre of the captured fish. Filling the knowledge gaps on the specific physiological and behavioural differences, for the different species used, will help us assess their welfare better and to select the appropriate welfare indicators. For the sustainability of the activity, the welfare of the non-targeted species should also be considered and evaluated. Moreover, focused welfare management in the different phases of CBA will be beneficial for both the animals and the farmers, lessening at the same time the environmental burden induced by CBA. The present review, did not explore the moral aspects of capturing and rearing wild fish. This is a very important issue and should be addressed in future studies, regardless of whether efforts are made to maintain a high level of welfare among the animals. However, it is part of a bigger discussion, concerning many other animal species. Therefore, as the discussion on the welfare of fish lagged behind, in relation to the discussion of the welfare of other animals, the discussion on the morality of the exploitation of captive wild fish will have to wait until decisions are made for many other animal species.

## Figures and Tables

**Table 1:** Summary of capture techniques, recovery phase and aquaculture stage of commonly used fish species in capture-based aquaculture. N/A indicates that data are not available.

Fish Species	Catching Size and Life Stage	Fishing Gears Used to Capture Seed	Mortality Rate of Wild Seed	Recovery Method	Aquaculture Method	Aquaculture Duration	Feed Type	Market Size	Areas Practiced	Ref.
Bluefin Tuna Northern Bluefin Tuna (*Thunnus thynnus*, *T. orientalis*) Southern bluefin tuna (*Thunnus maccoyii*)	Juveniles (100–300 g) of less than 8 kg. Sub adults and adults 20–80 kg, 80–120 kg, up to 600 kg).	Purse seines, tuna traps, mid-water trawls.	1–2% during transportation to grow out cages. Mortality rate at stocking about 15% During first month of adaptation mortality rate is 2.1%.	Slow speed towing cages, to avoid mortality due to lactic acid accumulation in muscles.	Circular/ring type open-sea floating net cages (diameter 30–50 m).	Farming: >20 months, or 3–4 years in net cages, Fattening: 1–7 months. Southern bluefin tuna: fattening-3–10 months.	Small pelagic species (chilled or frozen) including sardine, pilchard, herring, mackerel, squid etc.	30–70 kg	Northern bluefin tuna in Mediterranean countries, Canada, Mexico, Japan, the USA Sothern bluefin tuna in Australia.	[5,7,8,9,10]
Atlantic Cod (*Gadus morhua*)	Juveniles 3–10 g, sub adults 1–2 kg.	Seine nets (e.g., Danish seine and Scottish seine), traps, shrimp trawlers, longlines.	Less than 5%.	Special cages with a flat and taut bottom to recover from swim bladder rupture.	Floating cages.	6–8 months.	Artificial feed.	4–5 kg	Norway, Iceland.	[7,11]
European eel (*Anguilla anguilla*)	Glass eels or elvers <10 cm.	Stow nets, fyke net with a fine mesh-1 mm^2^), plankton nets, flow traps and dip or scoop nets (gear used by hand and gear pushed by a boat).	Could be high post-fishing.	Holding tank.	In freshwater, low-density flow-through pond culture under ambient conditions (<5–10 kg/m^3^). Semi-intensive pond and tank culture under semi-controlled conditions (10–100 kg/m^3^), super intensive recirculation tank culture in a completely controlled environment (>100 kg/m^3^) In brackish and marine water Mediterranean lagoons (“valli”)	6–24 months.	Live feed (e.g., artemia, worms) to wean glass eels and elvers Moist paste for glass eels and artificial dry feed for later stages.	200–500 g	Europe, Asia, Africa.	[5,7]
Japanese eel (*Anguilla japonica*)	Glass eels and elvers.	Scoop nets operated at night with a light, fine-meshed bag nets and elver traps.	Could be high post-fishing.	N/A	Outdoor ponds, basic greenhouse ponds, ponds with a bio-filtration unit or sedimentation unit.	N/A	Similar to European eels.	250–500 g	China, the Republic of Korea, Japan, Taiwan Province of China, Malaysia, Thailand.	[5,7]
Shortfin eel (*Anguilla australis australis*)	Glass eels and elvers.	Fine mesh fyke nets and dip nets.	Could be high post-fishing.	N/A	Pond-based culture includes extensive or intensive pond systems)	12–24 months.	Similar to European eels	150–300 g	Australia	[5,7]
Yellowtail/ Amberjack (*Seriola dumerili*, *Seriola lalandi* *Seriola quinqueradiata*)	Larvae 2–10 g Juveniles 25–100 g.	Seine nets, scoop nets, pair trawlers, traps.	5–10%	Nursery pens.	Net cages/enclosures.	About 8–12 months, or up to 3 years.	Fresh fish or artificial feed.	0.3–6 kg	Japan, Republic of Korea.	[7]
Groupers (*Epinephelus* spp.)	Fry (2.5–7.2 cm) fingerlings (7.5–12 cm).	Gango (fish nests) Fish shelters Miracle hole Fyke net Scissor net are destructive.	Using traps–3–5% Using fyke nets and scissor nets −30–70%.	Tanks or net cages or earthen ponds for a month or more (nursing period).	Earthen ponds Floating cages are often constructed from bamboo poles and polyethylene netting.	12–24 months	Low-value/Trash fish, Artificial feed.	1–2 kg	Southeast Asia, China.	[5,7]
Flathead grey mullet (*Mugil cephalus*)	Fry/fingerlings 10–15 g.	Fine seine nets, beach seine nets, scoop nets, hand nets.	High mortalities due to less acclimatization Without acclimatization–96% With acclimatization–6%.	Hapas or shore aggregation tank.	Traditionally practiced in the “hosha” extensive, semi-intensive ponds and netted enclosures.	About 7–8 months (one season), two successive seasons	Natural food by artificial fertilization, artificial feed.	0.75–1.75 kg	Mediterranean countries, Southeast Asia, Taiwan Province of China, Japan and Hawaii.	[7]
Indian major carps (*Catla catla*, *Labeo rohita*, *Cirrhinus mrigala*, *Labeo calbasu*)	Mixture of major and minor carp spawn.	Savar net (funnel shaped fixed net).	Mishandling may cause mass mortalities of spawn.	N/A	Ponds.	N/A	Natural food by fertilization and artificial feed in nursery ponds.	N/A	Bangladesh.	[7]
Milkfishes (*Chanos chanos*)	Larvae 1–2 cm.	Nets (e.g., push nets, dragged seine nets, fixed nets, hand nets, fry sweeper), longlines trap	10–20%.	Nursery ponds.	Ponds or cage and pen culture.	4–8 months.	Natural food by fertilization, low-value/trash fish, feed produced on site.	250–300 g	Philippines, Sri Lanka, Pacific Islands and Indonesia.	[7,12]
River catfish (or Sutchi catfish) (*Pangasianodon hypophthalmus)* Bocourt’s catfish (*Pangasius bocourti*)	Larvae (0.9–1.7 cm) Fry/fingerling (12–15 cm).	Specialized bagnets, or dais and stationery trawls, hooks with baits.	N/A	N/A	Earthen ponds or cages on tributaries of the Mekong River.	About 8 months.	Mainly commercial or home-made artificial feed.	1–1.5 kg	Vietnam, Cambodia.	[7]
Giant snakehead (*Channa micropeltes*), Chevron snakehead (*Channa* *striata*)	Fry/juvenile snakeheads.	Traps, cast-nets and lift-nets.	N/A	N/A	Cages, pens, rice fields.	N/A	Low-value/trash fish.	500–700 g	Vietnam, Cambodia, Lao People’s Democratic Republic.	[7]
Clarias catfish (*Clarias jaensis*, *Clarias gariepinus*)	Fingerlings 20–120 g.	Hand nets, seine nets, cast nets and baskets.	Sometimes about 90% due to cannibalism.	100 L containers, in earthen or concrete tanks for up to a month.	Earthen ponds, ponds stocked with Nile tilapia (*Oreochromis niloticus*) and flood ponds in Cameroon.	9–11 months in mixed culture with Nile tilapia 1–2 years in flood ponds.	N/A	N/A	Bangladesh, China, India, Indonesia, Malaysia, Philippines, Thailand, Cameroon.	[7]

**Table 2:** Suggested Operation Welfare Indicators for capture-based aquaculture (CBA) practices.

Phase of CBA	Suggested Operation Welfare Indicators			
	Animal Based		Environment Based	
	Individual Based	Group Based	Environmental Based	Housing/Rearing System Based
Capture phase	Superficial damages such as scale loss, tears of fins, surface wounds etc. Color change (e.g., glass eels become opaque).	Mortality.	Not applicable	Capture tools and equipment.
Recovery phase	Swimming and resting behavior (e.g., disoriented swimming, resting on bottom etc.). Response to feeding. Superficial damages.	Mortality.	Water quality parameters (temperature, salinity, dissolved oxygen, nitrogen wastes, etc.).	Stocking density (depending on the system, life stage, management practices, etc.).
Aquaculture phase	Condition factor. Feed conversion ratio. Disease related signs (e.g., disoriented swimming, fin erosion, emaciation etc.).	Mortality. Behavior (e.g., schooling).	Water quality parameters (e.g., temperature, salinity, dissolved oxygen, light).	Stocking density (as above).

## Data Availability

Not applicable.
